# Evidence from HIV sequencing for blood-borne transmission in Africa

**DOI:** 10.4102/jphia.v16i1.715

**Published:** 2025-04-30

**Authors:** David Gisselquist, Simon Collery

**Affiliations:** 1 Independent Researcher, Hershey, Pennsylvania, United States of America; 2 Borough of Camden, London, United Kingdom; 3 Independent Researcher, London, United Kingdom

**Keywords:** HIV, transmission, Africa, blood-borne, sequencing

## Abstract

**Background:**

The consensus view that heterosexual transmission dominates human immunodeficiency viruses (HIV) epidemics in sub-Saharan Africa survives side-by-side with surveys and studies reporting infections in children with HIV-negative mothers, in virgins, and in adolescents and adults who claim no possible sexual exposure to HIV.

**Aim:**

In this scoping review, we aim to show what phylogenetic analyses of HIV sequences say about the possible contribution of blood-borne transmission to HIV epidemics.

**Setting:**

The focus was on sub-Saharan Africa.

**Method:**

The authors conducted a search on PubMed and other platforms for studies reporting phylogenetic analyses of HIV in blood samples collected from at least 100 infected adults through community-based surveys in sub-Saharan Africa. They focussed on identifying information pertinent to assessing blood-borne transmission.

**Results:**

Sixteen reports met the search criteria and provided information to assess blood-borne transmission. In five studies, similar HIV sequences from (reported or assumed) household couples identified a likely heterosexual source for 0.3% – 7.5% of community adults with sequenced HIV. In 10 studies, a median of 43% of sequence pairs linked two people of the same sex. Two studies report clusters of recent infections too large to be easily explained by sexual transmission.

**Conclusion:**

Evidence from sequencing agrees with much other evidence that blood-borne HIV transmission is not rare in sub-Saharan Africa. Evidence also allows that blood-borne transmission could be making a major contribution to Africa’s HIV epidemics.

**Contribution:**

Evidence of harm is sufficient to stimulate discussions about what more could be done to address this continuing problem.

## Introduction

Human immunodeficiency virus (HIV) epidemics in sub-Saharan Africa have been more damaging than elsewhere, especially for women. According to United Nations Progarmme on HIV/AIDS (UNAIDS)’s estimates for end-2023, 5.7% of adults aged 15–49 years were living with HIV in Eastern and Southern Africa (ranging from 9.7% to 25.1% in eight countries) and 1.2% in Western and Central Africa, while only 0.3% of adults were infected in the rest of the world. Among adults aged ≥ 15 years in sub-Saharan Africa, almost twice as many women as men were infected (16.0 vs 8.7 million), whereas in the rest of the world, more than two men were infected for every woman (9.4 vs 4.5 million).^1^ According to UNAIDS’ estimates, men who have sex with men (MSM), including transgender women with similar risks, and injection drug users (IDUs) accounted for < 5% of incidence among adults aged 15–49 years in sub-Saharan Africa in 2022 compared to more than half outside the region.^2,3^ Much higher HIV prevalence in sub-Saharan Africa, especially among women, comes from the two remaining risks: sex between men and women and skin-piercing procedures other than IDU.

In 1990, the World Health Organization (WHO) circulated estimates that 1.6% of HIV infections in Africa and parts of the Caribbean through 1988 were from ‘inadequately sterilised skin-piercing instruments (health sector and outside)’.^4^ That estimate disagreed with the evidence available at the time from 20 studies of risks for prevalent and incident HIV infections in general population adults (including inpatients) in Africa. In those studies, average population-attributable fractions of HIV infections from skin-piercing risks (e.g. injections, transfusions, scarification and abortions) taken together exceeded those from sexual risks (e.g. sex worker contact, previous or current sexually transmitted disease [STD] and more than one sex partner)^5^; this evidence left unresolved questions about reverse causation (did medical procedures transmit HIV or treat conditions because of HIV infection?) and confound (was STD a marker for sexual risk or did treating it transmit HIV?).

In 1999, several WHO staff published a model-based estimate that medical injections annually infected as many as 100 000 people in sub-Saharan Africa with HIV.^6^ In 2004, WHO and UNAIDS staff led a team of 15 authors reaffirming the consensus: ‘there is no compelling evidence that unsafe injections are a predominant mode of HIV-1 transmission in sub-Saharan Africa’.^7^ Such estimates and assessments ignored HIV from other skin-piercing procedures such as infusions, dental care and tattooing, among others. As of 2025, the consensus focus on sexual transmission continues with supporting hypotheses, for instance, that sex workers and clients account for a lot of HIV infections (UNAIDS’ model-based estimate is 17% of adults’ incidence in 2022^2,3^) and that young women get HIV from sex with older men and then infect young men through sex.

Fortunately, good evidence is increasingly available to assess the contribution of blood-borne transmission to Africa’s epidemics. Studies that collect and sequence HIV from people in a community can say, with varying degrees of certainty based on similarities among sequences, whose infections are linked by direct or indirect (through one or more others) transmission. From large sequencing studies in sub-Saharan Africa, we report results relevant to assessing the contribution of blood-borne transmission to Africa’s epidemics. Most results come from sources published in 2017 or later. After 2013, the Phylogenetics and Networks for Generalised Epidemics in Africa (PANGEA) consortium assisted most studies.^8^

## Methods

This is a scoping review^9^ of evidence from phylogenetic analyses of HIV-1 sequences to assess blood-borne transmission in sub-Saharan Africa. We describe the protocol here; it is not otherwise published. We worked independently; D.G. did most of the searching, screening and data extraction, with S.C. assisting, advising and reviewing.

We looked for reports in any language and year in refereed medical journals; we accepted reports in grey literature if authors and phylogenetic analyses were linked to other reports in refereed medical journals. Because the sequencing evidence we wanted to review required large studies with teams of field workers along with analysts and computer experts, those who managed such studies would reasonably aim to publish what they found in refereed medical journals.

We searched for reports through PubMed, PANGEA’s list of published articles,^10^ arXiv, medRxiv, bioRxiv, Google Scholar, Social Science Research Network (SSRN) and our own files. In PubMed, we used the search terms: (hiv OR ‘human immunodeficiency virus’) and transmission and (cluster* OR network*) and (molecular OR phylogenetic) and (Africa). In arXiv, medRxiv, bioRxiv, Google Scholar and SSRN, we used the search terms ‘HIV Africa sequencing’ or ‘HIV Africa’.

We screened reports identified in our search for sources to include in this review with the following criteria:

- Inclusion criteria: studies that compared HIV sequences from at least 100 adults (to provide more reliable data) sampled from the general population through community-based surveys (including not strictly random surveys), with or without additional HIV samples collected from adults in or near the areas surveyed.
- Exclusion criteria: studies sequencing HIV samples from outside the surveyed area or from adults selected for high-risk behaviours (e.g. sex workers, MSM), except when such sequences can be excluded from reported sequence clusters.

After screening, we searched selected sources for evidence to identify or suggest the blood-borne or sexual risks that infected persons with sequenced HIV.

## Results

We searched PubMed on 12 May 2024 and updated our search on 02 February 2025 (see Preferred Reporting Items for Systematic Reviews and Meta-Analyses [PRISMA] flow diagram based on a template from reference^11^). PubMed returned 308 references (omitting one preprint listed later as a peer-reviewed article). From PANGEA’s February 2024 list of published articles, we found 17 more sources (after removing 13 duplicates). Screening sources from these two searches identified 32 that met our criteria. From other searches (after removing duplicates), we found seven more sources that met our criteria.^12, 13, 14, 15, 16, 17, 18, 19, 20, 21, 22, 23, 24, 25, 26, 27, 28, 29, 30, 31, 32, 33, 34, 35, 36, 37, 38, 39, 40, 41, 42, 43, 44, 45, 46, 47, 48, 49, 50^

Our criteria for accepting articles to review left us with some judgement calls. For example, we accepted a study^16^ that analysed samples from 233 HIV-positive adults in five fishing villages,^51^ along with 50 with incident infections in a follow-up cohort selected for sexual risk (including the very minor risk of being away from home at least two nights in a previous month)^52^; we considered that study to be based on a not strictly random survey that otherwise met our criteria. We also accepted some overlap; for example, we include two studies that sampled adults in the Rakai Community Cohort in 2008–2009 from 46 communities^14^ and in 2011–2014 from 40 communities.^15^

We searched these 39 sources for evidence to assess the contribution of blood-borne or sexual transmission to observed sequence clusters. Evidence of sexual transmission limits estimated infections from blood-borne risks. We found good information on sexual transmission within the home (see questions 1 and 2 in Table 1). Only one study identified any non-household sex partners with similar sequences (question 3 in Table 1). We looked for other evidence to assess the contribution of blood-borne risks to HIV transmission outside the home (questions 4–6 in Table 1): the percentages of sequence pairs linking same-sex or male-female subjects; physical distances between men and women with clustered sequences; and clusters of recent infections too large to be easily explained by sexual transmission.

**TABLE 1:** Sources with information to assess blood-borne human immunodeficiency virus transmission.

Questions	Sources with information	
	From PubMed search	Other sources
**Sexual transmission within the household**		
1. What percentage of infections can be explained by HIV-positive household sex partners?	Botswana;^12^ South Africa;^13^ Uganda^14,15^	Malawi^24^
2. What percentage of seroconcordant HIV-positive household sex partners have linked infections?	Uganda^14,15,16^	Malawi^24^
**Sexual and blood-borne transmission outside the household**		
3. What percentage of infections can be explained by identified HIV-positive non-household sex partners?	Uganda^17^	None
4. What percentage of sequence pairs (excluding, if possible, household sex partners) have same-sex and male-female members?	Kenya;^18^ Kenya and Uganda;^19^ South Africa;^20^ Uganda^15,16,17,21,22^	Botswana;^25^ Zambia^26^
5. Do the locations of non-household cluster partners agree with the reported or reasonable locations of non-household sex partners?	Uganda^14^	Botswana^25^
6. Do any clusters have 10 or more recent infections?	Cameroon^21^	South Africa^27^

Note: Please see the full reference list of the article, Gisselquist D, Collery S. Evidence from HIV sequencing for blood-borne transmission in Africa. J Public Health Africa. 2025;16(1), a715. https://doi.org/10.4102/jphia.v16i1.715, for more information.

Overall, 16 of 39 sources provided information to answer at least one question (Table 1).^12,13,14,15,16,17,18,19,20,21,22,23,24,25,26,27^ Three reviews^28,29,30^ repeated information from some of the sources in this review but provided no additional information. Information from one report^31^ was superseded by later information from another^12^ and 19 others answered none of the questions.^32,33,34,35,36,37,38,39,40,41,42,43,44,45,46,47,48,49,50^ Except for one conference presentation,^27^ all 16 sources used in this review are in refereed medical journals.

Studies used various methods to identify linked sequences. All studies looked for the most likely HIV transmission chains (hereditary or phylogenetic trees) among sequenced HIV, and most also considered specific measures of genetic distance (differences) between sequences. Beginning in 2019, some studies reported results from sequencing multiple quasi-species from each HIV sample (deep sequencing) and comparing multiple sequences from different samples. We accept clusters and linkages as reported. Along with results, we note how studies identified linked sequences.

### Sexual transmission in the home

#### Percentages of infections explained by household partners

Five of 16 sources collected all HIV samples from a comprehensive or random sample of households in a community, took blood from all cooperating adults in each household (not just one per household), and identified household sex partners (spouses or likely sex partners) in sequence clusters. These five studies identified HIV-positive household partners to explain 0.3% – 7.5% of adults with sequenced HIV in each community (Table 2).

**TABLE 2:** Identified and presumed household sex partners in sequence clusters.

Study community, years in which samples were collected	Adults with sequenced HIV	Seroconcordant couples with sequenced HIV	Number (%) of seroconcordant couples in clusters		Number (%) of sequenced adult infections explained by household sex		
			*n*	%	*n*	*N*	%
Mochudi, Botswana, 2010–2013^12^	833	NR	15†	-	15	833	1.8
Karonga, Malawi, 1981–1989^24^	174	17†	13†	76	13	174	7.5
uMkhanyakude, South Africa, 2011–2014^13^	1222†	NR	4†	-	4	1222	0.3
Rakai, Uganda, 2008–2009^14^	1099	105	51	49	51	1099	4.6
Rakai, Uganda, 2011–2015^15^	2652	331	176	53	176	2625	6.6

Note: Please see the full reference list of the article, Gisselquist D, Collery S. Evidence from HIV sequencing for blood-borne transmission in Africa. J Public Health Africa. 2025;16(1), a715. https://doi.org/10.4102/jphia.v16i1.715, for more information.

NR, not reported.

† , see paragraphs on household transmission in Botswana, Malawi, and South Africa (https://doi.org/10.4102/jphia.v16i1.715).

A study using blood samples collected during 2010–2013 from all cooperating adults aged 16–64 years in northeast Mochudi town, Botswana, looked for similarities among HIV sequences from 833 samples.^12^ The study found 322 sequences similar to one or more others (with strong statistical support for phylogenetic clustering of *env* genes and unspecified low genetic diversity), including 30 in 15 pairs from men and women living together. The study does not say they were sex partners. Assuming they were, one likely infected the other, providing a likely sexual explanation for 1.8% (*n* = 15/833) of Mochudi adults with sequenced HIV.

A study that tested blood samples collected from all cooperating residents in two regions of Karonga District, Malawi, during 1981–1989 identified 185 HIV-positive adults aged ≥ 15 years and two infected children. The study captured an emerging epidemic: the first HIV-positive sample was from 1982; in 1987–1989, 2% of adults were infected.^53^ The study was able to sequence HIV samples from 176 residents, including both spouses in 18 household couples.^24^ Thirteen couples had ‘closely related sequences consistent with transmission between spouses’ (the genetic distance between sequences was ≤ 2.0% in gag gene and ≤ 1% in *env* gene); four couples with less closely related sequences (the genetic distance between sequences was > 2.0% in *gag* gene and/or ≥ 5% in *env* gene) ‘may have been infected from different sources … but this would be surprising at a time when HIV prevalence was low’.^24^ The same genes were not sequenced for the partners in one couple and so could not be compared. Accepting authors’ initial sequence-based estimate and ignoring speculations based on low HIV prevalence, 13 couples with similar HIV sequences provide a sexual explanation for 7.5% (*n* = 13/174) of adults with sequenced HIV (assuming two of 176 sequences were from infected children).

A study that collected blood from all consenting adults aged 15–54 years in a study area in uMkhanyakude District, KwaZulu-Natal, South Africa, in 2011–2014 looked for similarities among HIV sequences from 1222 people with a known place of residence.^13^ The study found 333 sequences similar to one or more others (with high statistical support for phylogenetic linkage and < 4.5% genetic distance in *pol* gene), including four pairs of similar sequences from men and women living together and not more than 5 years apart in age. The study does not say they were sex partners. Assuming they were, the four pairs provide a likely sexual explanation for 0.3% (*n* = 4/1222) of adults with sequenced HIV and information on residence.

A study in Rakai District, Uganda, looked for similarities among HIV in blood samples collected in 2008–2009 from all cooperating adults aged 15–49 years in 46 communities.^14^ Sequences from 209 of 1099 adults clustered with one or more others (strong statistical support for phylogenetic clustering of *gag* or *env* genes with genetic distances of ≤ 1.3% in *gag* gene or ≤ 2.6% in *env* gene). Clusters linking partners in 51 stable couples provide a sexual explanation for 4.6% (*n* = 51/1099) of adults with sequenced HIV (one of 105 stable couples with sequenced HIV did not live in the same house; we group this couple with other household partners).

Another study from Rakai District, Uganda, deep-sequenced 2652 HIV samples collected in 2011–2015 from all cooperating adults aged 15–49 years in 40 communities. Deep sequencing found 1334 sequences in clusters, including 176 household couples with ‘highly supported male-female linkages’ providing a sexual explanation for 6.6% (*n* = 176/2652) of adults with sequenced HIV.^15^

#### Efficiency of sexual transmission between household partners

Three of five studies in Table 2 reported numbers of seroconcordant-infected household couples. In those three studies, HIV sequences from 49%, 53% and 76% of seroconcordant couples met the authors’ criteria to say one had likely infected the other.^14,15,24^ A fourth study that sequenced HIV in blood samples from five Ugandan fishing villages in 2009–2011 assessed that 58% (*n* = 7/12) of seroconcordant HIV-positive household partners had sufficiently similar sequences to say one had likely infected the other (with strong statistical support for phylogenetic clustering in *env* or *gag* genes and genetic distances ≤ 4.5% in *env* gene).^16^

To get an idea of the efficiency of sexual transmission between household partners, one has to consider the number of serodiscordant partners as well. None of the four studies reported that information. However, in a 2011 survey in Uganda’s Central 1 region, which includes Rakai District and four of five fishing villages in the third study, only 35% of household couples with HIV in one or both partners were infected.^54^ Hence, if the three Ugandan studies correctly identified 49%, 53% and 58% of couples with linked infections, less than a fifth (~35% × ~53%) of those who brought HIV into the home had infected their current partners.

#### Synthesising evidence of sexual transmission within the home

In five studies, similar HIV sequences in reported or presumed household sex partners identified a sexual source for 0.3% – 7.5% of community adults with sequenced HIV. Because these five studies, no doubt, missed many household partners (who refused to give blood or were not home, divorced or dead), and phylogenetic analyses may have missed some linked infections, percentages of infections from household sex partners may reasonably be several times greater. Even so, sexual transmission within the home appears to make no more than a minor to medium contribution to HIV epidemics in study communities. The estimate from Rakai data that less than a fifth of those who brought HIV into the home had infected their current partners is consistent with widely reported low rates of sexual transmission per coital event.

### Blood-borne and sexual transmission outside the home

#### Identified non-household sex partners

Only one article that met our search criteria identified any non-household sex partners in sequence clusters. That article reported a Ugandan study that sequenced HIV in blood samples from 74 adults recruited during community surveys and from 46 with incident infections in a community-based cohort with at least minor sexual risks (e.g. being away from home at least two nights in a previous month). The study identified non-household sex partners that could explain 2.5% (*n* = 3/120) of adults with sequenced HIV: three women and one man in a cluster of recent infections (less than 1.5% difference in *pol* genes) had previous sexual relationships.^17^

#### Sequence pairs

Ten studies reported numbers of sequence pairs with same-sex and male-female members (Table 3). Four studies used deep sequencing to identify linked pairs in larger clusters;^15,22,25,26^ for the other six studies, Table 3 reports two-person clusters only. Excluding reported and assumed household couples gives a better idea of HIV transmission outside the home. For example, among HIV from blood samples collected in Rakai, Uganda, during 2011–2015, 70% (*n* = 376/537) of all sequence pairs with highly supported phylogenetic linkages determined by deep sequencing were male-female; but after setting aside 176 pairs from household couples, only 55% (*n* = 200/361) were male-female.^15^ Data from 4 of the 10 studies in Table 3 exclude household couples (3 identified them, and 1 collected blood from one adult per household). Across these four studies, a median of 59% of pairs were same-sex. Across all 10 studies, including household couples in six, 10% – 86% (median 43%) of linked pairs were same-sex.

**TABLE 3:** Relative frequency of same-sex and male-female pairs.

Country	Years	Sequence pairs				Source of collected HIV samples
		Number	% MM	% FF	% MF	
**Excluding household couples**						
Kenya	2003–2005^18^	7†	29	57	14	Residents aged 13–34 years in random households in two communities (we accept the authors’ supposition that one MF pair living together was a couple)
South Africa	2014–2015^20^	168‡	11	48	41	One aged 15–49 years in random households in a study area in KwaZulu-Natal
Uganda	2009–2011^16^	22§	27	32	40	A total of 233 adults with prevalent HIV in five fishing villages; 50 with incident HIV in a follow-up cohort selected for sexual risks; excluding seven household couples
Uganda	2011–2015^15^	361¶	22	22	55	All adults in random households in 40 communities in Rakai, excluding 176 couples
**Including household partners**						
Botswana	2013–2018^25^	153¶	4	42	54	All cooperating adults aged 16–64 years in random or all households in 30 communities; adults seen at local health facilities
Kenya, Uganda	2013–2017^19^	10†	10	0	90	All aged ≥ 15 years in 32 communities
Uganda	2005–2010^17^	10†	0	30	70	Seventy-four adults in the Masaka Rural Clinical Cohort; 46 with sexual risks in five fishing villages
Uganda	2014–2016^21^	13†	15	15	69	A total of 255 adults in community-based studies in fishing villages, including some selected for sexual risk; 351 adults from nearby health facilities or testing campaigns
Uganda	2014–2017^22^	92¶	13	9	78	Adults from the General Population Cohort in Kalungu district, fishing villages and local health activities
Zambia	2013–2018^26^	801¶	11	30	59	One adult per random household in nine communities; several times more adults seen at local health facilities

Note: Please see the full reference list of the article, Gisselquist D, Collery S. Evidence from HIV sequencing for blood-borne transmission in Africa. J Public Health Africa. 2025;16(1), a715. https://doi.org/10.4102/jphia.v16i1.715, for more information.

FF, female-female; MF, male-female; MM, male-male.

† , Pairs with ≤ 1.5% difference in *pol* or *gag* genes;

‡ , Pairs with ≤ 4.5 differences in *pol* genes;

§ , Pairs with ≤ 4.5% differences in *env* genes;

¶ , Pairs identified by deep sequencing.

Most same-sex pairs linked two women. Anal sex among men could explain some male-male pairs; none of the studies reported such risks. Some same-sex pairs could be indirectly linked through sex with others of the opposite sex.

#### Do the locations of non-household cluster members agree with reported or reasonable locations of non-household sex partners?

Comparing the locations of non-household cluster members and reported or reasonable locations for non-household sex partners provides evidence to assess the likelihood that sexual or blood-borne transmission is linked to non-household cluster members. Two studies provide sufficient information to make such comparisons.

**FIGURE 1:**
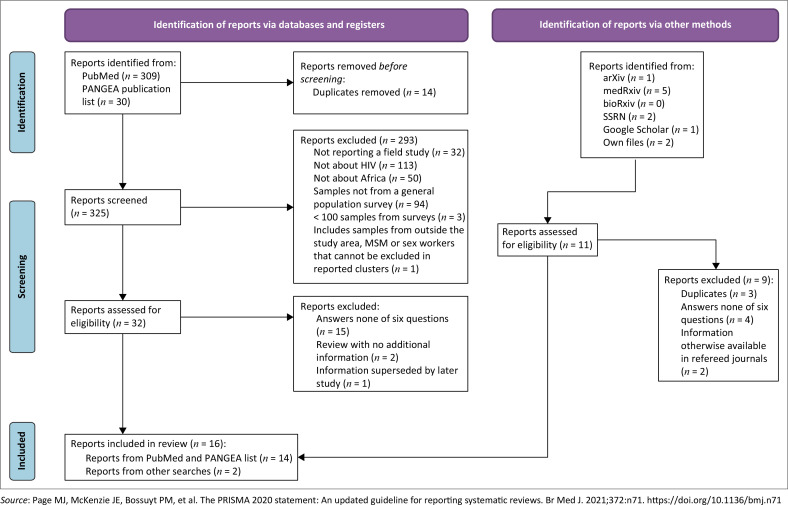
Preferred reporting items for systematic reviews and meta-analyses flow diagram for scoping review of evidence for blood-borne HIV transmission in Africa.

From HIV in blood samples collected in Rakai District, Uganda, 2008–2009, 72% (*n* = 38/53) of sequence clusters (strong statistical support for phylogenetic linkage of *gag* or *env* genes and genetic distances ≤ 1.3% in *gag* gene or ≤ 2.6% in *env* gene), including people from more than one household, linked people from two or more Rakai communities.^14^ However, only 28% (*n* = 929/3271) of reported non-household sex partners in the previous year lived in other Rakai communities (reported by 9520 adults who were HIV-negative or found with new infections in the 2008–2009 survey; see Figure 1 and Table S8 within reference Grabowski MK et al.^14^).

A study that deep-sequenced HIV in blood samples from 3832 adults in 30 communities across Botswana in 2013–2018 identified 25 ‘highly supported probable source-recipient [male–female] pairs’ linking people from different communities.^25^ These 25 pairs linked men and women living a median of 161 km apart; 1/4th lived at least 420 km apart.

#### Large clusters of recent infections

A study that sequenced HIV in blood samples collected from all consenting adults in a study area in KwaZulu-Natal, South Africa, identified a cluster of 63 very similar HIV sequences in samples collected from men and women in 2014. From similarities among sequences, researchers dated the last common ancestor for the 63 infections to mid-2013, thereby estimating that transmission to all cluster members took not more than 18 months.^27^ This was likely part of a much larger cluster: it was found in a study that sampled *circa* 15% of infected adults in the study area; it centred on a peri-urban area straddling the study area’s eastern boundary and likely extended outside the study area; and transmission may have continued after sample collection ended.

A study of HIV sequences from villages in southern Cameroon, 2011–2013, identified a ‘recent transmission’ chain (with less than 1.5% differences between *pol* genes) linking 10 women in 5 villages along a road.^23^

#### Synthesising evidence for the mode of transmission outside the home

Only one study identified any non-household sex partners in sequence clusters. Although no study identified any blood-borne risk to explain any sequence cluster, two studies reported large clusters of 10 and 63 closely linked infections that are difficult, if not impossible, to explain by anything other than IDU or other blood-borne risks. The high percentages of sequence pairs linking two men or two women suggest that the sex or whoever supplied the HIV transmitted outside the home was often a matter of chance (e.g. the last previous patient at a dental clinic) rather than sex. Several studies supposed, without presenting relevant behavioural evidence, that most or all same-sex pairs were linked by sexual transmission through untested partners. Similarly, all studies supposed all male-female pairs were linked through sexual transmission; but without evidence each such pair had a sexual relationship, that was an unwarranted assumption. In two studies, some of the clusters that linked men and women across long distances may be better explained by skin-piercing events at facilities (e.g. hospitals, dental clinics) drawing people from long distances rather than by long-distance sexual partnerships. Taken together, this is strong evidence that blood-borne transmission outside the home is not rare. This evidence also allows that blood-borne transmission could account for a large proportion, even a large majority, of transmission outside the home.

## Discussion

According to evidence assembled in this scoping review, household sex likely made not more than a minor to medium contribution to HIV infections in community adults. Only one study identified any non-household sex partners in sequence clusters, and no study reported any blood-borne risks to explain any sequence cluster. However, based on other evidence summarised in the previous paragraph, blood-borne transmission outside the home is almost certainly not rare. That agrees with much other evidence from surveys and studies; for instance, a 2015 national survey in Mozambique reported that 33% of HIV-positive children aged 6–23 months had HIV-negative mothers.^55^ Furthermore, the evidence considered in this review allows that blood-borne transmission could account for a large proportion, even a majority, of HIV transmission.

### Policy implications

From a policy perspective, the proportion of infections from blood-borne risks is not important and will in any case be debated and may be resolved over time. The policy challenge to the public health community and to local and national governments from this review (and from much other evidence) is to reconsider attention to blood-borne transmission and efforts to reduce it. UNAIDS’ 2024–2025 strategy for HIV prevention does not mention blood-borne transmission except IDU.^2^ On the other hand, the WHO’s strategy for HIV prevention during 2022–2030 identifies ‘people exposed through unsafe blood supplies and unsafe medical injections and procedures’ as a priority population for HIV prevention.^56^ To protect patients and clients, the WHO recommends standard precautions in the formal and informal health sectors and in cosmetic services. Because such recommendations have been in place for decades while the problem has continued, infection control and public health experts and governments may consider additional initiatives. Rich and poor countries around the world have for the most part contained nosocomial HIV transmission at very low rates, so strategies to do so have been demonstrated.

### Implications for research

Insofar as blood-borne transmission contributes to Africa’s epidemics, one would expect it to be more important among groups (e.g. sex workers and young women) with higher incidence. In this review, we looked for the best available evidence to assess blood-borne transmission, and so we did not select studies to look for such differences. Even so, we found evidence that blood-borne transmission may help to explain some observed epidemic patterns. One of the studies included in this review sequenced HIV samples collected from sex workers and clients in Kampala, Uganda, in 2014–2017. With deep sequencing, the study found 10 ‘source-recipient pairs [among sex workers] with strong support for phylogenetic linkage and direction of transmission’.^22^ This evidence suggests that skin-piercing healthcare or cosmetic services may be infecting sex workers in some venues. Similarly, although we did not look for evidence of blood-borne transmission to young women, the cluster of 10 recent infections among women in Cameroon suggests likely blood-borne transmission; the study did not report their ages.^23^

In 2014, Frost and Pillay advised that ‘the major focus for implementing molecular epidemiological approaches for HIV should be towards reducing the devastating epidemics in Africa and Asia’, and that ‘[i]t is critical for large sequence data sets to be placed side by side with detailed clinical, epidemiological, and behavioural information, to maximise the potential of phylogenetic approaches’.^57^ Significant efforts are still required to fully address that advice. For example, studies from Botswana and South Africa did not report if household couples were sex partners (we assumed they were). Similarly, with a few exceptions, studies did not report collected information (or did not collect it) on lifetime and non-spousal sexual partners, which would allow or disallow sexual transmission to explain clusters linking adults outside the home. Studies did not report (and likely did not collect) information on what skin-piercing risks participants received and where; in practice, it may be more efficient to collect such information in follow-up studies or investigations to find and stop specific skin-piercing risks after someone has recognised unexplained infections or clusters.

Some studies ignored their own evidence. For example, researchers who used deep sequencing to find highly supported phylogenetic linkages for 80 sequence pairs linking women in Rakai District, Uganda (see Table 3), considered those pairs to be errors because ‘HIV-1 is predominantly sexually transmitted in Africa’.^15^ Two later articles used only the male-female sequence pairs from that study to estimate the frequency of transmission to and from lakeshore hotspots and the ages of transmitting men and women.^35,38^ Similarly, another study that deep-sequenced HIV samples collected from the lakeshore and nearby inland communities in Uganda also ignored same-sex pairs when estimating transmission to and from hotspots and ages of transmitting men and women.^22^ By ignoring same-sex pairs, these studies did not use all available evidence to elucidate transmission flows.

### Limitations in this review

To make this review manageable, we screened for studies that analysed HIV sequences from at least 100 adults contacted through surveys among the general population. That limit left out smaller studies with relevant information, for instance, a study that analysed 68 sequences^58^ and another that analysed 94.^59^ From a previous review, evidence from these and other smaller studies generally agrees with the evidence considered here.^30^ Another limitation, mentioned in the previous paragraph, is that sequencing studies to date have not reported enough information on sexual behaviour and skin-piercing events to identify specific risks responsible for more than a few non-household sequence clusters.

## Conclusion

From the sequencing evidence considered in this scoping review, blood-borne transmission during healthcare and cosmetic services is almost assuredly not rare. Furthermore, the evidence allows that blood-borne transmission could account for a large proportion, and even a majority, of HIV infections in Africa.
